# Metals in Cancer Research: Beyond Platinum Metallodrugs

**DOI:** 10.1021/acscentsci.3c01340

**Published:** 2024-02-07

**Authors:** Angela Casini, Alexander Pöthig

**Affiliations:** † Chair of Medicinal and Bioinorganic Chemistry, Department of Chemistry, School of Natural Sciences, Technical University of Munich, Lichtenbergstraße 4, D-85748 Garching b. München, Germany; ‡ Catalysis Research Center & Department of Chemistry, School of Natural Sciences, Technical University of Munich, Ernst-Otto-Fischer Str. 1, D-85748 Garching b. München, Germany

## Abstract

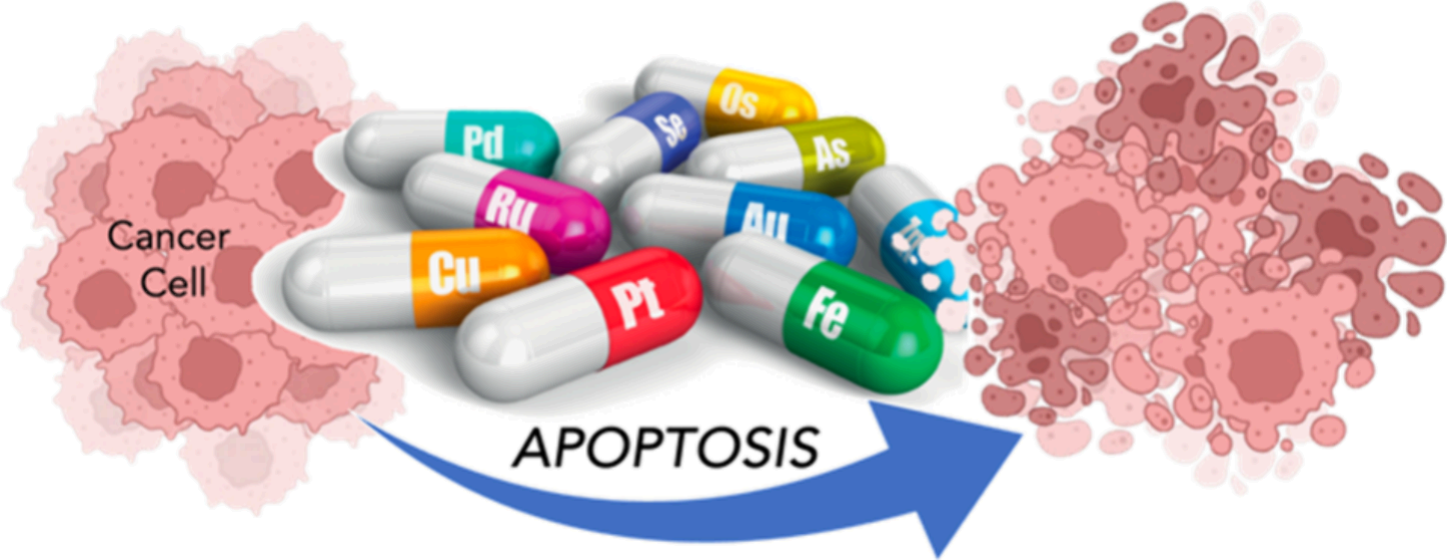

The discovery of the medicinal properties of platinum complexes has fueled the design and synthesis of new anticancer metallodrugs endowed with unique modes of action (MoA). Among the various families of experimental antiproliferative agents, organometallics have emerged as ideal platforms to control the compounds’ reactivity and stability in a physiological environment. This is advantageous to efficiently deliver novel prodrug activation strategies, as well as to design metallodrugs acting only via noncovalent interactions with their pharmacological targets. Noteworthy, another justification for the advance of organometallic compounds for therapy stems from their ability to catalyze bioorthogonal reactions in cancer cells. When not yet ideal as drug leads, such compounds can be used as selective chemical tools that benefit from the advantages of catalytic amplification to either label the target of interest (e.g., proteins) or boost the output of biochemical signals. Examples of metallodrugs for the so-called “catalysis in cells” are considered in this Outlook together with other organometallic drug candidates. The selected case studies are discussed in the frame of more general challenges in the field of medicinal inorganic chemistry.

## Abstract 2

The unique and exceptional features of metal-based compounds, markedly distinct from classical small-molecule organic drugs, are at the frontiers in the progress of cancer therapy and drug discovery.

## 1 Introduction

**Figure 1:**
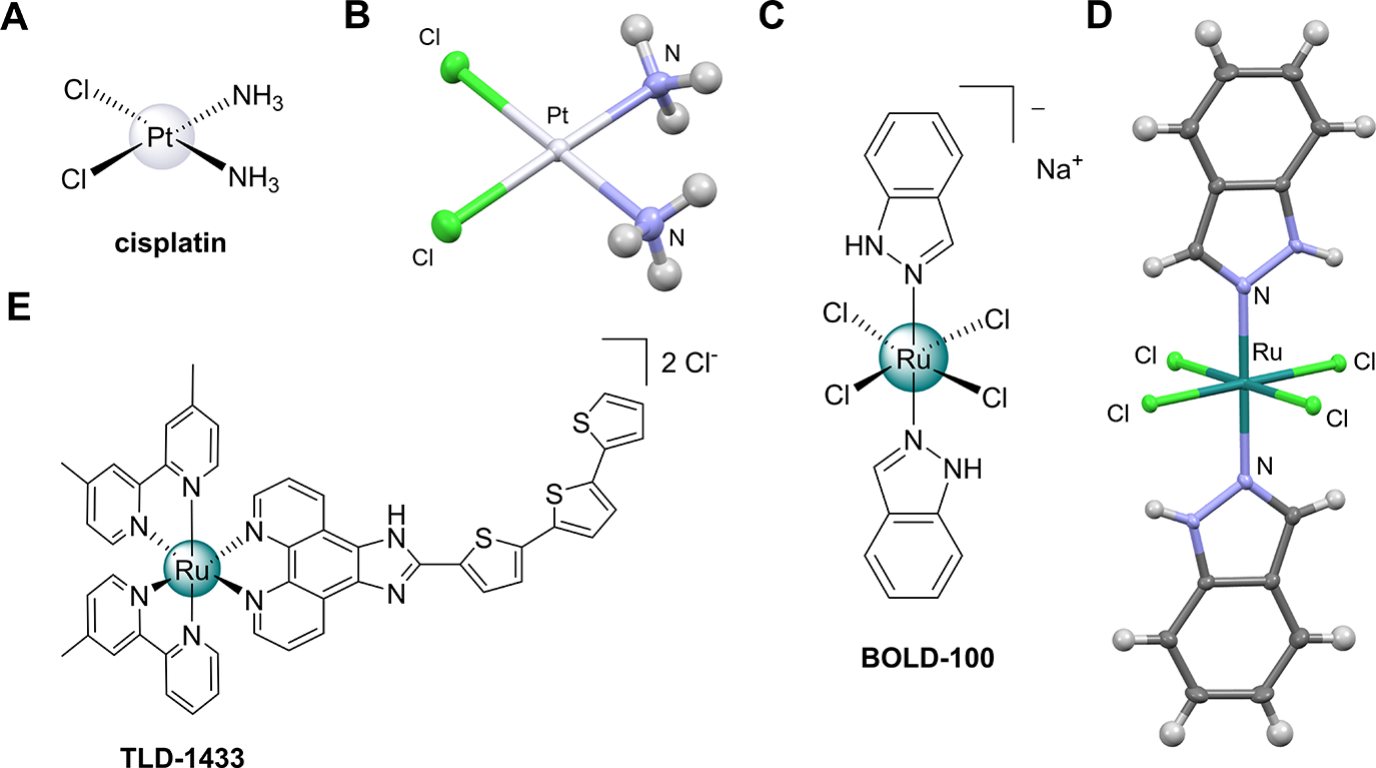
Structure of cisplatin (A) and its molecular structure (B) as determined by single-crystal X-ray diffraction (SC-XRD) taken from CCDC entry CUKRAB02. (C and E) Structures of Ru(II) compounds in clinical trials. (D) Molecular structure of BOLD-100 as determined by SC-XRD taken as an excerpt from the CCDC entry UFIDUJ. SC-XRD structures realized with Mercury (version 2023.2.0).

Medicinal inorganic chemistry involves the use of metal ions/metal-based compounds or metal ion binding components in a biological system for the treatment or diagnosis of diseases.^1−3^ While the contribution of metal-based compounds to the ensemble of therapeutics and imaging agents available in the clinic is extremely diverse and remarkable, the heartbeat of anticancer metallodrugs are the platinum(II) compounds, well-known before Alfred Werner classified coordination complexes,^4^ which have been studied intensely for several decades as cytotoxic agents. The most famous member of this family, cisplatin (*cis*-diamminodichloridoplatinum(II), Figure 1) was recognized as an anticancer drug in the late 1960s^5,6^ and was granted approval by the FDA in 1978. Used alone or in combination against different types of cancers, cisplatin is a blockbuster drug and one of the most successful therapeutic metallodrugs discovered so far. It is worth mentioning that, while for decades cisplatin’s mode of action (MoA) has been solely attributed to DNA distortion via Pt(II) binding to nucleobase residues, numerous studies point to the role of several proteins/enzymes in the compounds’ overall pharmacological and toxicological profiles, beyond classical serum transport proteins and metal detoxification systems.^7^

Following the discovery of the anticancer effects of cisplatin, the medicinal inorganic chemistry community has first focused on improving its pharmacological properties by changing the ligands at the Pt(II) center; this includes debating if only *cis* or even *trans* configurations would be suitable or varying the oxidation state to control the kinetics of prodrug activation. Additionally, others have moved to different metals in the transition series (e.g., Ru(III), Ir(III), Pd(II), Au(I), Ti(II), Cu(II), and Fe(II)),^8−15^ resulting in a completely different MoA and spectrum of antiproliferative activity. This first “holistic” approach was meaningful since it made the periodic table of elements a real toolbox of opportunities for researchers interested in anticancer drug development. This enthusiasm had to withstand the complexity of the reactivity of metal-based compounds in an aqueous/physiological environment, and often the lack of appropriate methods to investigate it in cellular contexts, which affected the understanding of the overall mechanisms of bioactivity and hampered the compounds’ optimization. A representative example of this first era of experimentation is the ruthenium complex sodium *trans*-[tetrachlorobis(1H-indazole)ruthenate(III)] (BOLD-100, former KP-1339, Figure 1) developed by Keppler and co-workers.^16−18^ Years of study and preclinical data have finally shown that the compound acts via a unique multimodal MoA. Like cisplatin, BOLD-100 causes DNA damage and cell cycle arrest,^19^ but it also induces apoptosis altering the unfolded protein response (UPR) through selective glucose regulated protein (GRP78) inhibition,^20^ a mechanism that appears difficult to evade and particularly effective against heavily mutated or resistant cancer cell lines. Moreover, histone deacetylation by BOLD-100 has been shown to play a pivotal role in the MoA.^21^ Overall, the compound has been identified as an epigenetically active substance acting via the targeting of several onco-metabolic pathways. Recent clinical data suggest that BOLD-100 could enhance the effectiveness of a wide range of existing cancer therapies and significantly improve patient outcomes. The compound has already received orphan drug designations (ODDs) in gastric and pancreatic cancer and is expected to receive a breakthrough therapy designation (BTD) in colorectal cancer and potentially other gastrointestinal cancer indications in the near future.^22^

In the last half-century, medicinal chemistry has advanced to the point where chemists can combine the principles of rationale design with high-resolution data from structural, spectroscopic, analytical and functional experiments to identify drug molecules with exquisite affinity and selectivity for even the most challenging of biological targets. For example, mass spectrometry techniques, both molecular and element-sensitive, hyphenated to include different separation methods, have been valuable investigational tools to provide information about the biological interactions of drug molecules at various levels. The case of electrospray ionization mass spectrometry (ESI-MS) is noteworthy since it is most suitable to study noncovalent “labile” interactions, such as coordination bonds between (bio)ligands and metal ions.^23^ Thus, proteomic/chemoproteomic and metallomics techniques have been extensively developed in the last decades to study metals in complex biological systems and to achieve target identification.^24,25^ These methodological advancements, together with a better understanding of the hallmarks of cancer, have benefited the field of metallodrug discovery, leading to more rational design concepts and targeted applications, as discussed in the next sections. As a consequence, a paradigm shift has occurred whereby a multitargeted approach in anticancer metallodrug design is preferable over a single target approach to overcome drug resistance mechanisms. In this context of mechanism-oriented anticancer drugs, a “hybrid” medicinal chemistry approach exploiting the unique behavior of transition metal complexes combined with clinically applied pharmacophores has provided, in some instances, significant advancement,^8,9,13,26−30^ not to mention the opportunities offered by the synergistic cooperation of heteronuclear complexes.^31,32^

While a large body of work has focused on second and third row transition metals due to their versatile chemical and photophysical properties, their intrinsic toxicity due to the possible interference with endogenous metal ion pathways and their limited availability have fostered research in first row transition metal complexes. The latter are also attractive since endogenous metal ions’ homeostasis and signaling contribute importantly to health and disease states, and their modulation or disturbance may lead to translational opportunities to leverage disease vulnerabilities. Without going into great detail, the cases of cuproplasia^33^ and ferroptosis^34^ are worth highlighting. The former is defined as copper-dependent cell growth and proliferation. This term encompasses both neoplasia and hyperplasia, describing the effects of copper in different signaling pathways, and includes both enzymatic and nonenzymatic copper-modulated activities. As such, cuproplasia can be pharmacologically targeted either via copper-selective chelators or copper ionophores,^35^ in addition to genetical or pharmacological disturbance of proteins involved in copper homeostasis. Instead, concerning ferroptosis, its hallmark is the iron-dependent lipid peroxidation, a phenomenon associated with cell and tissue demise, thus constituting a nonapoptotic form of cell death.^34^ Many studies have provided evidence that ferroptosis can inhibit tumor growth. Therefore, substances that regulate iron ions, such as chelators and regulators of related proteins or organelles, can control cancer progression via modulation of ferroptosis.^36^

In the arsenal of modern metallodrugs, the case of metal compounds as photosensitizers for photodynamic therapy (PDT) deserves a special mention.^2^ PDT is defined as the combination of a photosensitizer, light, and the production of singlet oxygen leading to Fenton/Fenton-like chemistry in cancer cells. The resulting oxidative stress results in cell death via apoptosis. PDT circumvents the problems of poor selectivity of classical chemotherapeutic treatments as the spatial and temporal activity of the metal complex can be precisely controlled through its activation by light. Coordination complexes that serve as effective PDT agents are composed of a metal ion with slow on- and off-kinetics, coordinated by ligands that produce accessible, long-lived, and reactive excited states. The successful example is represented by the Ru(II) polypyridyl photosensitizer TLD-1433 (Figure 1), which was the first Ru(II) complex to enter human clinical trials for PDT, designed from a tumor-centered approach, as part of a complete PDT package that included the light component and the protocol for treating nonmuscle invasive bladder cancer.^2^ Following successful phases 1–2, TLD-1433 has been designated fast track status by the FDA.

Finally, concerning metal compounds for application in nuclear medicine, the research efforts are aimed at the synthesis, characterization, and biological evaluation of target-specific metal-based radioactive probes for nuclear imaging (single photon emission computed tomography (SPECT) and positron emission tomography (PET)) or internal radiotherapy. The potential of therapeutic radiometals has recently been realized and relies on ionizing radiation (β^–^ and α) and Auger electrons to induce irreversible DNA and cellular damage, resulting in cell death. For example, molecularly targeted radiation therapy with lanthanide isotopes is now a viable alternative in treating neuroendocrine tumors (NETTER-1 trial, Lutathera, ^177^Lu-DOTA-TATE)^37^ and prostate cancers (VISION trial, Pluvicto, ^177^Lu-PSMA-617).^38^ Most notably, it is in this area of “metals in medicine” that the concept of *theranostic*, a treatment strategy that combines therapeutic and diagnostic approaches, has been successfully accomplished via different modalities.^39^

In this Outlook, we highlight new trends in anticancer metallodrug design, focusing on examples of therapeutic organometallic compounds at the early stages of preclinical investigation, as well as at the crossroad with other chemistry domains, such as catalysis and chemical biology. Specifically, we present the advantages of using organometallics not only to control metallodrug metabolism, but noticeably to either design noncovalent protein/DNA binders or to modify pharmacological targets via metal-templated reactions in cancer cells.

## 2 Anticancer Organometallic Drugs

One of the main issues with metallodrug design is the control of their *speciation* in a biological environment. Speciation for metal complexes includes ligand exchange reactions with biological nucleophiles, hydrolysis, geometric isomerization reactions, as well as redox reactions in the case of transition metals with different attainable oxidation states. If on one hand, these processes can prevent the metallodrug from reacting with its pharmacological targets, on the other hand, they can also contribute to its activation and bioactivity. Cisplatin is exemplary in this case, whereby the intact Pt(II) compound is a prodrug which undergoes activation upon hydrolysis of the chlorido ligands in cancer cells.^40^ Therefore, metal speciation can be beneficial but needs to be tightly controlled in time and space, through the body and across various cellular compartments, until the compound reaches its targets. In order to achieve such tuning and control of the compound’s reactivity and prodrug activation, aside the implementation of drug delivery systems,^41^ organometallic complexes have been proposed, whereby the presence of a direct metal–carbon bond usually enhances the kinetic stability of the compounds in physiological conditions. Thus, over the years various classes of organometallic compounds have been successfully assessed for their anticancer effects *in vitro* and *in vivo*. Among the most popular families, “piano-stool” metal arenes, metal *N*-heterocyclic carbene (NHC) complexes, cyclometalated, carbonyl, and alkynyl compounds have been widely explored.^42−47^ In particular, the NHC complexes can be assumed to dissociate from late transition metals very poorly and are therefore, also called “spectator ligands”.^48,49^ However, more recently the (metal-dependent) lability of the organometallic bond has been studied in greater detail,^50,51^ opening up new venues toward the design and application of organometallics in the biomedical context. In addition to their stability toward speciation, the adjustable robustness of the organometallic scaffold is also attractive for further functionalization of the compounds, for example, to achieve bioconjugation to targeting moieties^52,53^ (peptides, antibodies, nanoparticles, etc.) or to increase solubility and introduce fluorescent tags for cellular imaging.

### 2.1 Noncovalent Organometallic Binders

**Figure 2:**
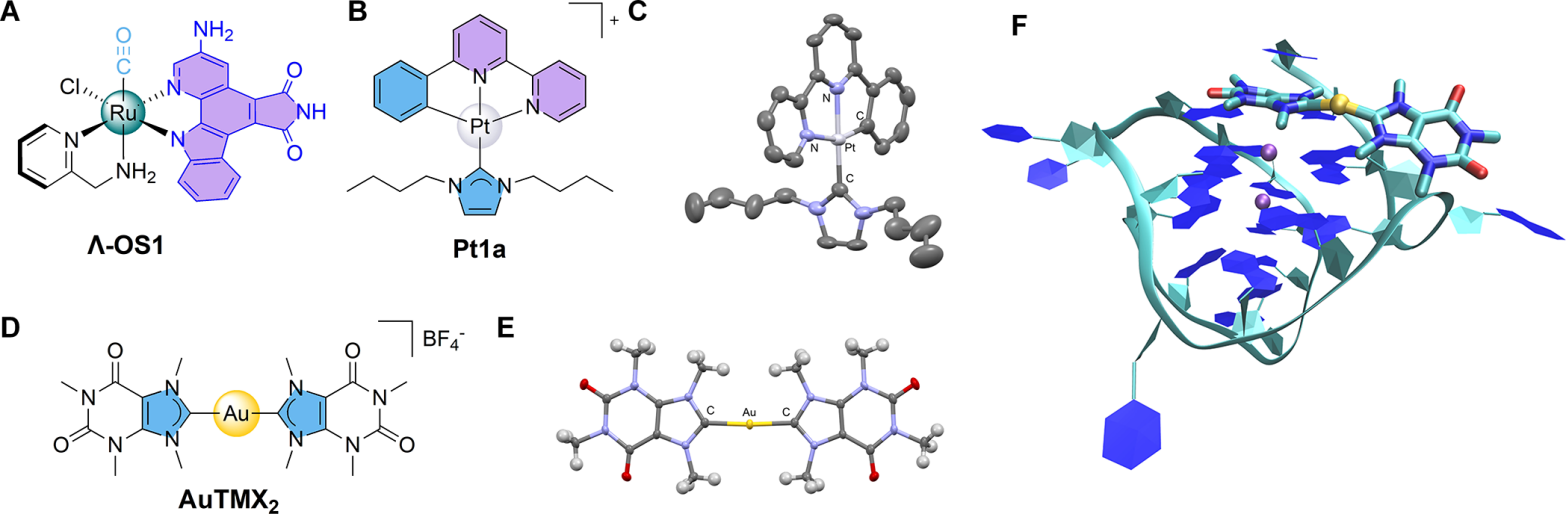
(A) Structure of the monocarbonyl Ru(II) kinase inhibitor **Λ-OS1** with the staurosporin-inspired binding domain in violet; (B) structure of the cyclometalated Pt(II) complex [Pt(C^∧^N^∧^N)(NHC_2_Bu)]PF_6_ (**Pt1a**; HC^∧^N^∧^N = 6-phenyl-2,2′-bipyridine; NHC^2Bu^ = *N*-butyl substituted heterocyclic carbene); (C) molecular structure of **Pt1a** as determined by SC-XRD, taken from the CCDC entry IPIYIQ (ellipsoids shown at 30% probability, hydrogen atoms omitted for clarity). (D) Structure of the Au(I) NHC complex **AuTMX**_**2**_ and (E) molecular structure of the **AuTMX**_**2**_ cation as determined by SC-XRD, taken from the CCDC entry YENBAW. SC-XRD structures realized with Mercury (version 2023.2.0). (F) Noncovalent adduct of **AuTMX**_**2**_ with the promoter G4 structure cKit-1 calculated by multiple collective variable (CV) metadynamics.^66^ G4s color scheme: sugar backbone = turquoise, DNA bases = blue, potassium ions = purple spheres. Compound **AuTMX**_**2**_ in stick representation, color scheme: carbon = turquoise, nitrogen = blue, oxygen = red, hydrogen = white, Au(I) = yellow sphere. Figure generated with VMD software.

Binding of metal complexes to pharmacological targets can be achieved in different ways: either by direct coordination of the metal center or noncovalently by supramolecular interactions of the organic ligands with the target. In general, for most of the reported examples of anticancer metallodrugs, the two binding modes coexist, and more or less stable coordination bonds with biological nucleophiles (e.g., amino acid residues, nucleobases, etc.), as well as electrostatic, hydrophobic, π–π, and H-bonding interactions, can be established. Thus, many examples of cytotoxic organometallic complexes as DNA binders have been reported.^54−56^ As a noticeable example of compounds acting purely via noncovalent interactions with their proteinaceous targets, we praise the Ru(II) complexes developed by Meggers and co-workers as kinase inhibitors using the indolocarbazole alkaloid staurosporine as a lead structure.^57^ For example, in the monocarbonyl Ru(II) kinase inhibitor **Λ-OS1** (Figure 2A),^58^ the coordinative and organometallic bonds are introduced to be kinetically inert in biological environment. In such a stable octahedral coordination sphere, the metal can be considered as a virtually hypervalent carbon providing untapped opportunities for the design of novel three-dimensional (3D) molecular structures, which populate previously inaccessible regions of chemical space in enzyme pockets via noncovalent bonding. Following this pioneering concept, Cohen and co-workers have implemented the use of “inert” metallofragments as 3D scaffolds for fragment-based drug discovery (FBDD).^59^ A more recent example of organometallic complexes acting via noncovalent interactions with protein targets features the antitumor and antimetastatic cyclometalated Pt(II) complex [Pt(C^∧^N^∧^N)(NHC^2Bu^)]PF_6_ (**Pt1a**; HC^∧^N^∧^N = 6-phenyl-2,2′-bipyridine; NHC^2Bu^ = *N*-butyl substituted heterocyclic carbene; Figures 2B and 2C)^60^ that engages vimentin, a canonical biomarker of the epithelial–mesenchymal transition, fitting into a pocket between the coiled coils of the rod domain of vimentin with multiple hydrophobic interactions.

Noncovalent and preassociative intermolecular forces are essential also in nucleic acid recognition by metallodrugs and have been the subject of intense investigation using a variety of methods.^61^ In this context, the cationic caffeine-based bis-NHC Au(I) complex [Au(9-methylcaffeine-8-ylidene)_2_]^+^ (**AuTMX**_**2**_; Figures 2D and 2E) has emerged as a very effective and selective stabilizer of noncanonical nucleic acid structures, namely G-quadruplex (G4) DNA, which regulate telomere homeostasis, gene transcription, and DNA replication.^62^ Therefore, stabilization of G4s by small molecules, including metal complexes,^63^ may induce anticancer effects due to the resulting inhibition of telomere extensions or oncogene expression.^64^ Structural characterization of the binding modes of **AuTMX**_**2**_ with different G4s was achieved by both X-ray diffraction studies^65^ and atomistic simulations (Figure 2F),^66^ evidencing the importance of π–π stacking and possibly electrostatic interactions in stabilizing the Au(I) compound/G4 adducts. Interestingly, machine learning (ML) approaches have been implemented to accelerate metadynamic simulations and free-energy calculations using **AuTMX**_**2**_/G4 adducts as model systems.^67^ This work paves the way to key applications of ML in drug discovery, enriching the toolbox of methods available for computer-aided drug design (CADD) beyond quantitative structure–activity relationship (QSAR) analysis, virtual screening, and *de novo* drug design. Further validation of the multimodal MoA of **AuTMX**_**2**_ via noncovalent interactions has been achieved by shot-gun proteomics in ovarian cancer cells.^68^

### 2.2 Catalysis in Cells

In the last decades, organometallic compounds have attracted increasing attention not only for their kinetic stability and relative lipophilicity but also since they are amenable to previously unattainable chemical transformations in biological environments. In fact, the incorporation of abiotic transition metal catalysts into the chemical biology space has significantly expanded the number of bioorthogonal reactions accessible for *in vitro* and *in vivo* applications.^69,70^ In this context, catalytic metallodrugs are very attractive since they can achieve high efficiency at low dosages and overcome cancer cell resistance through novel MoA. The so far investigated catalytic systems, based on different metals, can not only initiate redox processes, but also perform many other types of transformations in living conditions, including transfer hydrogenation (TH) and cross-coupling reactions, cycloadditions, as well as functional group deprotection (uncaging) reactions.^71−81^

**Figure 3:**
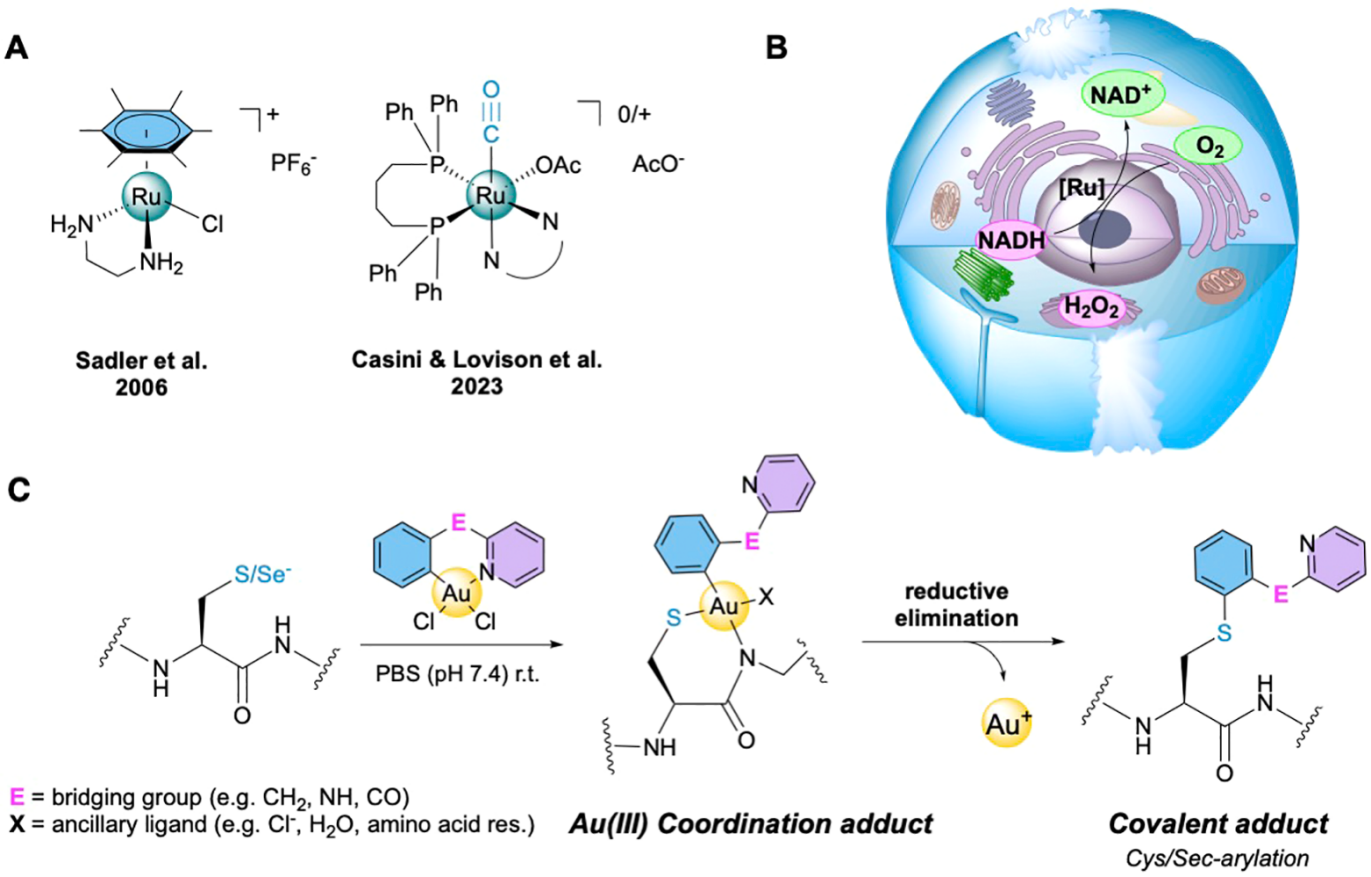
(A) Representative examples of organometallic Ru(II) complexes studied for transfer hydrogenation (TH) reactions in cancer cells. (B) Cartoon representation of the TH reactions leading to 1,4-NADH oxidation in cells catalyzed by organometallic Ru(II) compounds in the presence of molecular O_2_. (D) Scheme of the reaction of Au(III) cyclometalated compounds, featuring C^∧^N-type ligands, with cysteine or selenocysteine residues. Following the formation of a coordination adduct of the Au(III) center with the amino acid (Cys or Sec), the reaction can proceed toward C–S cross-coupling via reductive elimination in physiological conditions.

In 2006, following the pioneering work of Steckhan et al.^82^ on the rhodium(III) [Cp*Rh(bipy)Cl]^+^ (bipy = 2,2′-bipyridyl) compound capable of regioselectively restoring 1,4-NADH in aqueous media (pH 7, 37 °C) in the presence of formate as a hydride source, Sadler and co-workers proposed to exploit the TH properties of Ru(II)–arene complexes of the general formula [(η^6^-arene)Ru(en)Cl]PF_6_ (en = ethylendiamine; Figure 3A) to regenerate 1,4-NADH in cells.^83^ Using this strategy, the concentration of NAD^+^ as well as the NAD^+^/NADH ratio could be altered (Figure 3B), potentially interfering with numerous processes highly controlled in cancer cells, such as energy regulation, DNA repair and transcription, or immunological functions.^84^ Since these initial studies, a number of different Ru(II), Rh(III), Ir(III), and Os(II) organometallic compounds have been studied for either the regeneration of NADH or its oxidation in physiological conditions and/or *in cellulo*, and their antiproliferative activities have been determined.^73,75^ Some of these compounds are also able to catalyze the reduction of pyruvate to lactate using formate as a hydride source under biologically relevant conditions. Pyruvate is an important intermediate in metabolic pathways and the end product of glycolysis in cells and is ultimately destined for transport into mitochondria as a pivotal fuel input sustaining the Krebs cycle.^85^ Hence, the disturbance of pyruvate metabolism by metallodrug catalysts is expected to generate metabolic disorder.

While most of the organometallic drugs shown to perform TH in cells feature metal–arene moieties, we have recently reported on a different family of water-soluble organometallic TH catalysts based on a Ru(II) monocarbonyl scaffold of the general formula [Ru(OAc)CO(dppb)(N^∧^N)]^*n*^ (*n* = +1, 0; OAc = acetate; dppb = 1,4-bis(diphenylphosphino)butane; N^∧^N = different bidentate nitrogen ligands; Figure 3A).^86^ This class of Ru(II) compounds has some advantages over the classical Ru–arenes, including high versatility of the whole octahedral coordination environment at the metal center, offering more opportunities for catalyst design and functionalization, as well as possibilities to tune its chemico-physical properties. In general, for all these families of TH catalysts, it is unlikely that the reaction will work in optimal catalytic conditions due to metal detoxification mechanisms in cancer cells (e.g., glutathione (GSH)). Further catalyst design should aim at increasing the compounds’ stability with respect to inactivation by intracellular nucleophiles and at introducing targeting moieties (e.g., bioconjugation to targeting peptides). Alternatively, a few strategies have been proposed to tame GSH in living systems which could be implemented at early stages of metallodrug design.^87^

Another example of metal-templated reactions in cells is provided by the case of organometallic Au(III) compounds.^74^ In 2017, Tanaka and co-workers reported the first *in vivo* study on a gold-templated reaction, whereby a cyclometalated gold compound, exploiting the Lewis acid character of Au(III) ions, was capable of activating propargyl ester functions for binding to proteins by amide bond formation.^88^ Of note, we and others observed that bidentate C^∧^N-cyclometalated gold(III) compounds, following coordination to target cysteine residues in proteins, can template the formation of covalent aryl–peptide adducts via C–S cross-coupling (Figure 3C).^89−92^ The same arylation reaction can be achieved with selenol groups of selenocysteine residues. The reactivity of a Au(III) C^∧^N complex has been recently studied by combined chemoproteomic and proteomic approaches in cancer cells, and for the first time the unambiguous modification of selenocysteine by gold-templated arylation was observed *in cellulo*.^93^ Notably, the chemoproteomic data evidenced thioredoxin reductase (TrxR) as the main interactor for the Au(III) C^∧^N complex, further validating the role of this selenoenzyme in the MoA of cytotoxic gold complexes. Based on these results, gold(III)-templated reactions for covalent targeting of amino acid residues hold great promise for anticancer applications.

## 3 Conclusions and Outlook

In conclusion, we highlighted here recent strategies in the area of metallodrug development which we consider particularly intriguing, also from a mechanistic perspective, based either on catalytic pathways or purely relying on noncovalent interactions of organometallic compounds with different pharmacological targets. While the use of catalytic organometallic drugs holds promise to achieve controlled prodrug activation, alternative strategies have been developed, including stimuli-responsive activation; for example, via tumor associated stimuli (pH activity, redox variation, etc.), or photoactivation, which are equally valuable. We refer the readers to more comprehensive recent reviews for such approaches.^94−96^

Similarly, in the nuclear medicine domain, the emerging field of supramolecular radio-theranostics should also be mentioned, in which the classical radiopharmaceutical design is revisited and implemented by self-assembly strategies.^97−101^ The latter generate nanostructures via a wide range of noncovalent forces, including hydrophobic and electrostatic interactions, coordination bonds, and hydrogen bonding. As such, this approach is not only facile and flexible, but enables the synthesis via self-assembly of supramolecules with diverse topology and potentially unlimited *functionalizability*. Furthermore, via careful tuning of the binding kinetics and thermodynamics, these entities can give rise to the temporally and spatially controlled release of the active species. In this area, a new class of cationic supramolecular organometallic complexes (SOCs),^97^ named *pillarplexes*,^102^ has recently been reported to bind open DNA four-way Holliday junctions, creating exciting possibilities to modulate and switch such structures in biology.^103^ In general, based on these intriguing results, we expect that supramolecular approaches in medicinal inorganic chemistry will foster further attention.

Finally, in an attempt to identify outstanding challenges in our community, we reflect upon the importance of noncovalent interactions in metallodrugs’ reactivity. Indeed, many of the investigated metallodrugs, featuring coordination or organometallic bonds, exhibit their anticancer properties after coordinative bonding with their biological target(s), based on the principle of hard and soft acids and bases (HSAB theory). However, several examples have shown that the metallodrugs’ kinetic reactivity at the target site can also be highly affected by the surrounding microenvironment. The latter is mostly determined by the local structure of the biological target (e.g., the protein isoelectric point, pH, hydrogen bond, van der Waals and electrostatic interactions, dielectric constant of the binding pocket, etc.). A representative example is the case of carboplatin, which was designed to manifest extreme kinetic inertness at physiological pH to overcome the side effects of cisplatin and is today among the most important platinum(II) anticancer drugs. The compound, while showing generally scarce reactivity with protein targets, was proven to efficiently bind via ligand exchange reactions with methionine residues in the model protein cytochrome c, affording cisplatin-like adducts over time, as assessed by ESI-MS.^104^ This and many other examples demonstrate that, when exploring the metallodrug–biomolecule interactions, the fundamentals of inorganic coordination/organometallic chemistry must not only be applied but also adapted. Therefore, new chemical guidelines have to be defined based on a deeper understanding of metallodrugs’ interactions in biological systems, including taking into account confinement effects.^105−107^ In our opinion, this is one of the greatest challenges of modern medicinal inorganic chemistry, which may add new trends to the periodic table aimed at predicting the reactivity of metal compounds in physiological environment.
